# Supplementary material for: Meta-Analysis and Gene Set Enrichment Relative to ER Status Reveal Elevated Activity of MYC and E2F in the “Basal” Breast Cancer Subgroup
Source: PLoS One. 2009 Mar 9;4(3):e4710. doi: 10.1371/journal.pone.0004710 (PMC2650420; doi:10.1371/journal.pone.0004710)
Supplement: Dataset S1 — GSEA results from first screen. The file contains a zipped archive of the HTML results of the second GSEA screen. Reports for enriched or depleted datasets may be accessed from the “index.html” link within the my_analysis.GseaPreranked.1219975950625.rpt.zip ZIP archive. Enrichment plots are available for each of the 50 most enriched and depleted gene sets. (13.32 MB ZIP) [file pone.0004710.s001.zip › my_analysis.GseaPreranked.1219975950625.rpt.zip!//OLDAGE_DN.html]

Details for gene set OLDAGE\_DN[GSEA]

|  || Dataset | zSco.ERneg.to.ERpos\_collapsed |
| Phenotype | NoPhenotypeAvailable |
| Upregulated in class | na\_pos |
| GeneSet | OLDAGE\_DN |
| Enrichment Score (ES) | 0.6668671 |
| Normalized Enrichment Score (NES) | 2.3867185 |
| Nominal p-value | 0.0 |
| FDR q-value | 0.0 |
| FWER p-Value | 0.0 |
Table: GSEA Results Summary

  

Fig 1: Enrichment plot: OLDAGE\_DN      
 Profile of the Running ES Score & Positions of GeneSet Members on the Rank Ordered List

  

| PROBE | GENE SYMBOL | GENE\_TITLE | RANK IN GENE LIST | RANK METRIC SCORE | RUNNING ES | CORE ENRICHMENT || 1 | CDC20 |  |  | 9 | 7.151 | 0.0443 | Yes |
| 2 | FOXM1 |  |  | 21 | 6.760 | 0.0859 | Yes |
| 3 | CENPA |  |  | 51 | 6.315 | 0.1234 | Yes |
| 4 | CCNA2 |  |  | 52 | 6.297 | 0.1630 | Yes |
| 5 | NASP |  |  | 59 | 6.201 | 0.2015 | Yes |
| 6 | KIF2C |  |  | 124 | 5.703 | 0.2326 | Yes |
| 7 | KIF14 |  |  | 221 | 5.215 | 0.2581 | Yes |
| 8 | CENPF |  |  | 268 | 5.028 | 0.2862 | Yes |
| 9 | CTSC |  |  | 300 | 4.932 | 0.3149 | Yes |
| 10 | CDC25B |  |  | 340 | 4.826 | 0.3423 | Yes |
| 11 | RANBP1 |  |  | 380 | 4.733 | 0.3691 | Yes |
| 12 | CKS1B |  |  | 416 | 4.638 | 0.3956 | Yes |
| 13 | MYBL2 |  |  | 418 | 4.629 | 0.4246 | Yes |
| 14 | NUP88 |  |  | 475 | 4.451 | 0.4484 | Yes |
| 15 | APPBP1 |  |  | 565 | 4.213 | 0.4682 | Yes |
| 16 | MCM2 |  |  | 630 | 4.080 | 0.4890 | Yes |
| 17 | IL8 |  |  | 692 | 3.946 | 0.5092 | Yes |
| 18 | KIF11 |  |  | 713 | 3.909 | 0.5322 | Yes |
| 19 | FBN2 |  |  | 862 | 3.683 | 0.5442 | Yes |
| 20 | H2AFX |  |  | 930 | 3.591 | 0.5618 | Yes |
| 21 | PLK1 |  |  | 988 | 3.499 | 0.5795 | Yes |
| 22 | DDX39 |  |  | 989 | 3.498 | 0.6014 | Yes |
| 23 | UBE2C |  |  | 994 | 3.489 | 0.6231 | Yes |
| 24 | KIF23 |  |  | 1030 | 3.442 | 0.6421 | Yes |
| 25 | PTGS2 |  |  | 1202 | 3.232 | 0.6495 | Yes |
| 26 | PSMC2 |  |  | 1381 | 3.058 | 0.6553 | Yes |
| 27 | PKMYT1 |  |  | 1715 | 2.753 | 0.6475 | Yes |
| 28 | CKAP5 |  |  | 1738 | 2.731 | 0.6630 | Yes |
| 29 | CSE1L |  |  | 1990 | 2.537 | 0.6601 | Yes |
| 30 | H2AFZ |  |  | 2105 | 2.447 | 0.6669 | Yes |
| 31 | PARP1 |  |  | 2571 | 2.161 | 0.6454 | No |
| 32 | PSMD11 |  |  | 2726 | 2.077 | 0.6469 | No |
| 33 | BARD1 |  |  | 3028 | 1.920 | 0.6363 | No |
| 34 | CCNF |  |  | 3265 | 1.809 | 0.6299 | No |
| 35 | HADH2 |  |  | 3873 | 1.565 | 0.5940 | No |
| 36 | HMGB2 |  |  | 3883 | 1.558 | 0.6031 | No |
| 37 | HAS2 |  |  | 5204 | 1.079 | 0.5104 | No |
| 38 | ATR |  |  | 5834 | 0.867 | 0.4685 | No |
| 39 | PSMD12 |  |  | 5876 | 0.852 | 0.4707 | No |
| 40 | PSMA2 |  |  | 6855 | 0.533 | 0.4004 | No |
| 41 | PSMA3 |  |  | 7737 | 0.218 | 0.3354 | No |
| 42 | SAFB |  |  | 7777 | 0.198 | 0.3337 | No |
| 43 | PPP1CC |  |  | 8722 | -0.180 | 0.2637 | No |
| 44 | POSTN |  |  | 10398 | -1.089 | 0.1444 | No |
| 45 | PSMC6 |  |  | 11776 | -2.298 | 0.0551 | No |
| 46 | CDH11 |  |  | 12438 | -3.164 | 0.0252 | No |
| 47 | UGCG |  |  | 13297 | -6.552 | 0.0017 | No |
Table: GSEA details [plain text format]

  

Fig 2: OLDAGE\_DN: Random ES distribution      
 Gene set null distribution of ES for **OLDAGE\_DN**
